# Conservation Strategies in the Genus *Hypericum* via Cryogenic Treatment

**DOI:** 10.3389/fpls.2016.00558

**Published:** 2016-04-27

**Authors:** Katarína Bruňáková, Eva Čellárová

**Affiliations:** Institute of Biology and Ecology, Faculty of Science, Pavol Jozef Šafárik University in KošiceKošice, Slovakia

**Keywords:** slow cooling, vitrification, cold acclimation, ABA, meristems, freezing tolerance, oxidative stress, hypericins

## Abstract

In the genus *Hypericum*, cryoconservation offers a strategy for maintenance of remarkable biodiversity, emerging from large inter- and intra-specific variability in morphological and phytochemical characteristics. Long-term cryostorage thus represents a proper tool for preservation of genetic resources of endangered and threatened *Hypericum* species or new somaclonal variants with unique properties. Many representatives of the genus are known as producers of pharmacologically important polyketides, namely naphthodianthrones and phloroglucinols. As a part of numerous *in vitro* collections, the nearly cosmopolitan *Hypericum perforatum* – Saint John’s wort – has become a suitable model system for application of biotechnological approaches providing an attractive alternative to the traditional methods for secondary metabolite production. The necessary requirements for efficient cryopreservation include a high survival rate along with an unchanged biochemical profile of plants regenerated from cryopreserved cells. Understanding of the processes which are critical for recovery of *H. perforatum* cells after the cryogenic treatment enables establishment of cryopreservation protocols applicable to a broad number of *Hypericum* species. Among them, several endemic taxa attract a particular attention due to their unique characteristics or yet unrevealed spectrum of bioactive compounds. In this review, recent advances in the conventional two-step and vitrification-based cryopreservation techniques are presented in relation to the recovery rate and biosynthetic capacity of *Hypericum* spp. The pre-cryogenic treatments which were identified to be crucial for successful post-cryogenic recovery are discussed. Being a part of genetic predisposition, the freezing tolerance as a necessary precondition for successful post-cryogenic recovery is pointed out. Additionally, a beneficial influence of cold stress on modulating naphthodianthrone biosynthesis is outlined.

## Introduction

The genus *Hypericum* encompassing nearly 500 species is one of the most diverse plant genera in the angiosperms (Nürk and Blattner, 2010). The representatives of the genus are distributed throughout nearly all continents with an exception of the poles, deserts, and low-altitude tropical areas (Robson, 1996). Among them, *H. perforatum* L. is a perennial herb native to Europe, originally used as a folk remedy for the treatment of depression. The ‘Saint John’s wort’ became a subject of the British Herbal Pharmacopoeia (1996), the American Herbal Pharmacopoeia (1997), and the European Pharmacopoeia (2008) representing the most important and commercially recognized species of the genus *Hypericum*. Several groups of bioactive natural products involving naphthodianthrones (e.g., hypericin and pseudohypericin), phloroglucinols (e.g., hyperforin and adhyperforin), flavonol derivatives (e.g., isoquercitrin and hyperoside), biflavones, xanthones, proanthocyanidins, amino acids, and essential oil constituents have been identified in the crude drug of *H. perforatum*, Hyperici herba (Nahrstedt and Butterweck, 1997).

In the context of traditional medicine, recent pharmacological research confirmed anti-depressive activity and dermatological applications of *H. perforatum* extracts based on their anti-microbial (Saddiqe et al., 2010) and anti-inflammatory (Wölfle et al., 2014) effects. Recently, the naphthodianthrones hypericin and pseudohypericin have received most of the attention due to their antitumour (Penjweini et al., 2013) and antiviral (Arumugam et al., 2013) action. These compounds are concentrated in the clusters of specialized cells, so-called ‘dark nodules’ distributed on the leaves, stems, petals, sepals, stamens and ovules of many *Hypericum* taxa (Crockett and Robson, 2011). In plants, hypericin and its congener pseudohypericin are present mainly in protoforms which convert to their naphthodianthrone analogs upon activation by visible light (Rückert et al., 2006). It has been reported that the biosynthetic potential of *Hypericum* plants grown in outdoor conditions depends on environmental factors, mainly temperature and water stress (Gray et al., 2003; Zobayed et al., 2005). Therefore, development of *in vitro* culture systems for perspective biotechnological applications is indispensable.

In addition to the clonal multiplication procedure designed for *H. perforatum* (Čellárová et al., 1992), the *in vitro* systems involving both, other wide-spread cosmopolitan, and endemic *Hypericum* species have been established for *H. erectum* (Yazaki and Okuda, 1990)*, H. canariense* (Mederos Molina, 1991), *H. brasiliense* (Cardoso and de Oliveira, 1996)*, H. balearicum, H. glandulosum, H. tomentosum, H. maculatum, H. olympicum*, and *H. bithynicum* (Kartnig et al., 1996), *H. foliosum* (Moura, 1998), *H. patulum* (Baruah et al., 2001)*, H. androsaemum* (Guedes et al., 2003), *H. heterophyllum* (Ayan and Cirak, 2006), *H. polyanthemum* (Bernardi et al., 2007), *H. hookerianum* (Padmesh et al., 2008), *H. mysorense*, (Shilpashree and Ravishankar Rai, 2009), *H. frondosum, H. kalmianum*, and *H. galioides* (Meyer et al., 2009), *H. triquetrifolium* (Karakas et al., 2009; Oluk and Orhan, 2009), *H. retusum* (Namli et al., 2010)*, H. rumeliacum, H. tetrapterum, H. calycinum* (Danova, 2010)*, H. richeri* ssp. *transsilvanicum, H. umbellatum* A. Kern. (Coste et al., 2012), *H. cordatum* (Vell. Conc.) N. Robson (Bianchi and Chu, 2013), etc.

While the advances in the tissue culture techniques enable breeding of plants outside their natural habitat, genetic and epigenetic alterations increasing the potential of somaclonal variability in course of serial sub-culturing may occur (Kaeppler et al., 2000). To provide a more reliable method for saving rare or endangered taxa, the cryogenic storage represents a safe and long-term conservation opportunity for the plant specimens. In principle, the plant parts are stored in liquid nitrogen (LN) below the glass transition temperature (Tg) at which the cell solution forms an amorphous solid or glass. Under these conditions, the sample is biologically inert and can be maintained indefinitely (Bajaj, 1995; Butler and Pegg, 2012). Nevertheless, the viability of cells, tissues and organs is retained and regeneration of plants is acquired after the rewarming.

Despite an extensive research has been exerted in the course of total synthesis and semi-synthesis of hypericin (Huang et al., 2014), numerous *in vitro* studies indicate that shoot cultures of *Hypericum* spp. remain a reliable source of hypericin and other unique constituents. Concurrently, various cryopreservation techniques have been successfully applied to several *Hypericum* species maintaining the genetic features and biosynthetic capacity in the regenerated shoot tissues. Therefore, the aim of this review is to summarize advances in long-term conservation of *Hypericum* species by cryopreservation, and to analyze the relation between endo- and exogenous preconditions and post-cryogenic recovery and ability to synthesize unique bioactive substances.

## Cryopreservation Approaches and Post-Cryogenic Recovery in *Hypericum* Spp.

The earliest cryopreservation study of *H. perforatum* was carried out by Kimáková et al. (1996) who used the encapsulation-dehydration procedure. Isolated apical meristems were encapsulated in calcium alginate beads, osmoprotected with sugar solutions, partially dehydrated by exposure to a flow of dry air and directly immersed into LN. The first protocols resulted in a low, up to 10% survival (Kimáková et al., 1996), and the need for a more efficient long-term storage method for *H. perforatum* has arisen.

Later both, the controlled cooling and vitrification-based techniques were adopted for the cryoconservation of *Hypericum* spp. In principle, the controlled (slow) cooling method is based on crystallization induced in the extracellular solution, thus the probability of intracellular ice formation is minimized (Karlsson and Toner, 1996). Generally, the plants or their parts are pre-cultured under special conditions, such as low but above-freezing temperature, treated with growth regulators and/or osmotically active compounds, and exposed to cryoprotectants. Subsequently, the explants are subjected to slow cooling rates reaching the homogenous ice nucleation at -35 to -40°C and plunged into LN (Benson, 2008). After cryostorage, the samples are thawed rapidly in a 40 to 50°C water bath, and the cryoprotective chemicals are removed from the system by dilution. Usually, the incubation of explants in 1.2 mol L^-1^ sucrose for 10 to 20 min at room temperature is used (Shibli et al., 2006). On the other hand, the vitrification procedure performed by a direct immersion of the specimen into LN (so-called ‘rapid cooling’) is based on the complete elimination of ice formation throughout the entire sample (Karlsson and Toner, 1996). The protocols are based on cell dehydration performed by a standard sequence of steps involving: (i) exposure of the explants to diluted vitrification solutions such as loading solution (LS; Nishizawa et al., 1993), (ii) dehydration of the tissues performed by highly concentrated mixtures of cryoprotective agents, mostly plant vitrification solutions like the PVS2 or PVS3 (Nishizawa et al., 1993), (iii) direct immersion into LN, and (iv) rapid re-warming of the specimens followed by unloading phase at which the cryoprotectants are washed out of the cells.

Applying the controlled cooling for cryopreservation of *H. perforatum*, the isolated shoot tips were pre-treated with mannitol or abscisic acid (ABA), loaded in a mixture of cryoprotectants containing 10% (w/v) glycerol, 20% (w/v) sucrose, and 10% (w/v) ME_2_SO and exposed to gradual decrease of temperature (Urbanová et al., 2002, 2006). Cooling was performed in the programmed freezer up to -40°C followed by immersion into LN. The recovery after re-warming varied between 10 and 50% depending on genotype. Using the modified cooling regime by Skyba et al. (2011), the mean recovery varying in the interval from 0 to 34% was positively influenced by lowering the cooling rate.

According to the vitrification protocol published by Skyba et al. (2010), *H. perforatum* shoot tips were exposed to two different additives, either sucrose or ABA. Subsequently, the explants were loaded with LS and transferred to the cryovials filled with the PVS2 or PVS3. The samples were equilibrated on ice and immersed into LN. The post-cryogenic survival was strongly influenced by the genotype varying in the range from 0 to 62%. However, the highest mean recovery rate of 27% was recorded for the explants treated with ABA and subsequently exposed to PVS3. A comparably extensive variation of the mean recovery of *H. perforatum* shoot tips cryoconserved by a vitrification-based method was observed by Petijová et al. (2012). Despite a significant genotype-dependent variation, the post-cryogenic survival linearly increased in relation to extension of the pre-culture time. For instance, the prolongation of incubation of ABA-treated *H. perforatum* shoot tips in PVS3 resulted in an increased mean regeneration percentage reaching the maximum between 59 and 71% (Bruňáková et al., 2011).

Beside the ‘model’ *H. perforatum*, the vitrification protocol published by Skyba et al. (2010) was adopted for cryopreservation of *H. rumeliacum*, a species restricted to the Balkan region (Danova et al., 2012), and further optimized for several *Hypericum* species of different provenances involving both, cosmopolitan and endemic representatives. The post-cryogenic variation in the regeneration rate of *H. humifusum* L., *H. kalmianum* L., *H. annulatum* Moris., *H. tomentosum* L., *H. tetrapterum* Fries., *H. pulchrum* L., *H. kouytchense*. Lév., *H. canariense* L., and *H. rumeliacum* Boiss., was in the interval from 0 to 26% corresponding well with the inter-specific variability in the tolerance against freezing stress (Petijová et al., 2014).

For *H. richeri* ssp. *transsilvanicum* and *H. umbellatum*, the rare species found in Transylvania, a droplet-vitrification procedure was designed by Coste et al. (2012). Combining a ME_2_SO-droplet method and vitrification, less time for cryoprotection of the explants in a very small volume of cryoprotectant mixture is needed and substantially higher rate of cooling is achieved (Schäfer-Menuhr et al., 1997; Sakai and Engelmann, 2007). The post-thaw recovery depended on the type of explant, sucrose concentration in the pre-culture medium, and dehydration duration. The highest mean post-cryogenic recovery was obtained for axillary buds reaching 68 and 71% for *H. richeri* and *H. umbellatum*, respectively.

Moreover, the slow cooling and vitrification methods were successfully applied for undifferentiated cell suspensions of *H. perforatum* in order to find a possible relation between the ability of cryoprotective mixtures to decrease temperature of crystallization (TC) and post-cryogenic viability of the cells (Mišianiková et al., 2016). Among 13 cryoprotectant mixtures, the highest portion of viable cells exceeding 58% was reached in *H. perforatum* cell suspensions cryoprotected with a mixture containing 30% (w/v) sucrose, 30% (w/v) glycerol, 5% (w/v) ME_2_SO, and 20% (w/v) ethylene glycol and subjected to a controlled cooling. The results revealed that the highest cell viability correlated well with the lowest TC.

Although the genotypic effects may have contributed to the broad variation in post-thaw survival of *Hypericum* spp., the regrowth capability of cryopreserved meristems and cell suspensions was predominantly influenced by the pre-cryogenic sample preparation, mainly by the type and duration of the pre-culture, cryoprotection and rate of cooling.

## Crucial Processes for Successful Cryopreservation in *Hypericum* Spp.

The efficient cryopreservation protocol comprises series of procedures which enable the meristematic tissues to maintain the viability and regeneration potential at the freezing temperatures. Several processes have been recognized to be essential for post-cryogenic survival of *H. perforatum.* Among them, modifications of anatomical, morphological, and physiological status of the shoot apices in relation to the current views on structural changes occurring in the meristematic cells during preconditioning, and freezing-induced dehydration and phase transitions during cooling are further discussed.

### Effects of Preconditioning on Morphology, Anatomy, and Physiology of Shoot-Tip Meristems

The optimal preconditioning of plants or their parts is crucial for post-cryogenic survival and commonly includes chemical pre-treatments with exogenously applied growth regulators, osmotically active chemicals such as saccharides or saccharide alcohols, or subjection to cold acclimation prior to cryopreservation. Among the plant growth regulators, ABA is involved in mediation of many physiological processes including adaptation responses to environmental conditions comprising dehydration, osmotic, and cold stresses (Chandler and Robertson, 1994). The increasing level of endogenous ABA was observed under dehydration stress and cold treatment performed by the exposure of *in vitro* grown *H. perforatum* plants to subfreezing temperature of -4°C (Bruňáková et al., 2015). The phytohormone ABA is known to induce freezing resistance in many winter annual and perennial species (Bravo et al., 1998) and was shown to contribute to acquisition of the tolerance to cryopreservation, e.g., in *Triticum aestivum* L. (Chen et al., 1985), *Bromus inermis* Leyss., *Medicago sativa* L. (Reaney and Gusta, 1987), *Daucus carota* L. (Thierry et al., 1999), etc. Despite the exogenously applied ABA did not improve the resistance of *H. perforatum* shoot tips against freezing, the 3.5-fold higher level of endogenous ABA was observed in the freezing-tolerant *H. perforatum*, when compared with freezing-sensitive *H. canariense* (Bruňáková et al., 2015).

The pre-treatment with ABA is routinely used in numerous plant cryopreservation protocols (Buritt, 2008; Lu et al., 2009). In *Hypericum* cryoprotection, ABA is obviously used alone (Skyba et al., 2012) or in combination with sucrose or mannitol (Coste et al., 2012; Danova et al., 2012; Petijová et al., 2012). In addition to morphological alterations of the apical meristems expressed by an increased size of the meristematic domes (Petijová et al., 2012), a significant dehydration effect of ABA during preconditioning of *H. perforatum* shoot tips has been observed. Pre-treatment with ABA substantially affected total water content in the cryoprotected shoot apices. When compared to the hydration level of explants excised from cold acclimated plants, the shoot tips isolated from non-acclimated control group that was pre-cultured with ABA displayed a significantly lower amount of both, the total water content and the proportion of so-called ‘freezable water’ that can crystallize (Bruňáková et al., 2011). According to Danova (2010) and Danova et al. (2012), the extended period of ABA pre-treatment positively influenced the physiological state of *H. rumeliacum* apical meristems by decreasing the level of oxidative stress which consequently improved the status of plants regenerated after cryopreservation.

Despite ambiguous interactions between the plant hormone ABA and cytokinins have been reported in higher plants (Reed, 1993; Baldwin et al., 1998; Tran et al., 2007; Werner and Schmülling, 2009), the supplementation of media with cytokinins is usually used for micropropagation of plant material prior to cryopreservation and for increasing the proportion of proliferating meristems (Helliot et al., 2002). In *Hypericum* spp. cryopreservation, shoot tips are isolated from individually growing *Hypericum* plants or clusters of shoots developing on BA-enriched medium (Urbanová et al., 2006; Coste et al., 2012; Danova et al., 2012; Petijová et al., 2012). However, a long-acting influence of BA may reduce the post-cryogenic survival by alterations of morphological and physiological status of meristematic tissues (Petijová et al., 2012).

The longitudinal sections of *H. perforatum* shoot apices isolated from clusters revealed the meristematic domes uncovered with the leaf primordia which normally protect the proliferating cells from injurious effects of cryoprotectants. On the other side, the normal position of the first pair of leaves, compactness of apical meristem and increased mitotic activity in meristematic regions have been attributed to a synergic effect of ABA and BA during the short-term pre-culture of shoot tips before cryoprotection treatment (Petijová et al., 2012). In contrast, long-term culturing of the donor plants on media enriched with BA negatively influenced survival of shoot tips after cryopreservation. The results indicate that long-term exposure of plants to BA can delay the protective effect of ABA during preconditioning; the reduced tolerance to low temperatures may be attributed to the morphological alterations of the shoot tip meristematic regions isolated form BA-induced clusters of shoots (Petijová et al., 2012). At the physiological level, ABA and BA antagonized each other influencing the hydration status of *H. perforatum* meristems during preconditioning; while a massive accumulation of water was observed in the shoot tips solely pre-cultured in liquid BA-enriched media, the pre-treatment of explants with ABA resulted in a subsequent dehydration (Bruňáková et al., 2011).

Along with the phytohormone ABA, the saccharides are involved in freezing tolerance (FT) and are often used for preparation of plant tissues before cryopreservation (Dereuddre and Tannoury, 1995; Cho et al., 2000; Pâques et al., 2002; Shatnawi et al., 2011). Exogenously applied sucrose is known to stimulate the saccharide metabolism (Gibson, 2000), stabilize the native conformation of biomembranes (Kent et al., 2010), and reduce the proportion of freezable water which is important for minimizing the injurious effects of ice formation (Fang et al., 2009). Besides, the saccharides and saccharide alcohols prevent cryoinjury by increasing the osmotic pressure and reducing the size of treated cells (Cho et al., 2000). In *Hypericum* cryopreservation, pre-treatment of the explants in media supplemented with high concentration of sucrose was proved to be essential for post-cryogenic survival of *H. richeri* and *H. umbellatum* (Coste et al., 2012). In this work, incubation of the shoot apices or axillary buds in PVS2 without any previous saccharide pre-treatment of the explants did not provide enough protection from the lethal effects of LN. Using sucrose and mannitol as the pre-culture agents improved the post-thaw survival of *H. perforatum* shoot tips (Urbanová et al., 2002; Skyba et al., 2010, 2011; Coste et al., 2012; Petijová et al., 2012).

Another way in which the cryoprotectant agents relate to restoration of the viability after cryostorage is preservation of the photosynthetic apparatus. The protective roles of ABA and mannitol were confirmed on cellular and sub-cellular levels by transmission electron microscopy (TEM) in *H. perforatum* and *H. rumeliacum* plants (Stoyanova-Koleva et al., 2013, 2015). The effect of ABA depended on the species, duration of the treatment and cooling regime; in *H. perforatum*, although no deleterious post-cryogenic alterations of the internal membrane system under 10-day pre-treatment with ABA followed by slow-cooling were seen, the destruction of the chloroplast membranes was observed after vitrification (Stoyanova-Koleva et al., 2015). In *H. rumeliacum* shoot tips cryopreserved by vitrification, an optimal efficacy of the ABA-treatment was already observed after 3-day pre-culture (Stoyanova-Koleva et al., 2013). The positive influence of the saccharide alcohol mannitol was represented by the native structure of chloroplasts with a typical thylakoid arrangement in the palisade parenchyma cells in post-cryogenic *H. perforatum* regenerants (Stoyanova-Koleva et al., 2013).

Natural resistance to low temperatures can also be induced by subjection of plants to low but above-freezing temperatures in the process of cold acclimation (Thomashow, 1999). Cold-induced tolerance to freezing is effective in the preparation of plant meristems before cryopreservation, and was successfully applied for preconditioning treatment of the species originated from temperate regions such as *Cynodon dactylon* L. or *Allium sativum* L. (Volk et al., 2004; Reed et al., 2006). As a consequence, the anatomical and physiological changes of the tissues induced by cold-acclimation contributed to overall plant tolerance to cryogenic temperatures (Thinh, 1997). It has been observed that the exposure of freezing-tolerant *H. perforatum* or *H. rumeliacum* to a temperature of 4°C could entirely substitute the effect of ABA treatment during the pre-cryogenic phase (Bruňáková et al., 2014). These conditions resulted in almost 45% recovery in *H. perforatum* and led to 1.3- and 1.5-fold increase in the content of ABA in *H. perforatum* and *H. canariense* (Bruňáková et al., 2015). As a part of a cold-acclimation response, the elevated level of endogenous ABA is in agreement with a slight and transient increase of the hormone level in the model *Arabidopsis thaliana* (Lang et al., 1994).

Apart from the external factors that were shown to improve the cold tolerance of several *Hypericum* species, the physiological status of meristematic tissues *in vitro* was significantly influenced by genetically predetermined endogenous processes. The results of Skyba et al. (2011) predicted an existence of seasonal biorhythm altering the capability of *H. perforatum* shoot tips to regenerate after cryopreservation. In this study, the recovery rates depended on the season when the cryopreservation of *in vitro* initiated seedlings was performed; the nearly fourfold higher post-thaw regeneration favored the shoot tips cryopreserved in March in comparison with October.

### Freezing-Induced Dehydration and Phase Transitions during Cooling

Apart from the preconditioning, the cooling step represents a component of cryopreservation protocol having a significant influence on post-cryogenic cell survival. In the slowly cooled systems, the explants are exposed to specific cooling rates ranging from 0.1 to 5.0°C min^-1^ when ice is primarily initiated in the extracellular space. The optimal rate of freezing is a vital point of this process at which the damaging osmotic effects are minimized and the mechanical destruction of the cell organelles induced by intracellular crystallization is prevented (Kartha and Engelmann, 1994). Before subjection to freezing, the tissues are dehydrated by incubation in media supplemented with highly concentrated osmoregulants such as sucrose. However, for retaining the post-cryogenic viability, the plant organs should contain an optimal content of water varying from 0.25 to 0.4 g H_2_O g^-1^ dry weight (DW; Vertucci et al., 1991; Dereuddre and Kaminski, 1992; Wesley-Smith et al., 1992).

The analyses performed by differential scanning calorimetry (DSC) showed that the amount of freezable water in the shoot tips of *H. perforatum* which were cryoprotected with the mixture consisting of 10% (w/v) glycerol, 10% (w/v) ME_2_SO and 0.5 mol L^-1^ sucrose for 60 min remained high referring to 52% (3.05 g H_2_O g^-1^ DW; Skyba et al., 2011). In the partially hydrated biological systems such as seeds or embryos containing more than 0.25 g freezable H_2_O g^-1^ DW, the cooling velocity is known to critically affect post-cryogenic survival (Liebo and Mazur, 1971; Vertucci, 1989). Lowering the rates of cooling in the interval from 0.1 to 2.0°C min^-1^, the post-cryogenic recovery of *H. perforatum* shoot tips increased, reaching maximum of 34% at 0.3°C min^-1^ (Skyba et al., 2011). At the lower cooling rates, the improved survival of *H. perforatum* meristems corresponded well with the significantly higher compactness of cryopreserved apical domes when compared to disintegrated meristematic tissues of the shoot tips cooled at higher velocities. Based on the analyses of thermal gradients in the cryovials, the positive effect of slower cooling predominantly consisted in more homogenous temperature distribution in the sample during freezing resulting in a moderate and non-invasive growth of ice crystals.

The positive influence of the prolonged dehydration interval related to the lower rates of cooling was also confirmed for mesophyll cells of *H. perforatum* plants regenerated after cryopreservation (Stoyanova-Koleva et al., 2013). TEM analyses revealed the increasing protective effect of mannitol at lower cooling velocities. The pre-treatments of *H. perforatum* with 0.3 mol L^-1^ mannitol followed by the cryoprotection and cooling at 0.2°C min^-1^ resulted in a sustentative ultrastructure of the chloroplasts and other organelles in post-cryogenic regenerants.

On the other hand, cryopreservation of plants by vitrification completely eliminates formation of ice inside and outside the cell by combination of dehydration and rapid cooling. The critical viscosity of cytoplasm, at which the ice nucleation is inhibited and an amorphous, glassy solid is formed, is achieved by subjection of the explants to highly concentrated mixtures of cryoprotectants at non-freezing temperatures (Sakai et al., 1991). Although the precise mechanism by which the cryoprotectant compounds prevent the cells from freezing injury remains ambiguous, the loading time is an essential prerequisite for post-cryogenic recovery. It is necessary to apply an adequately long period of time to achieve sufficient dehydration and penetration of cryoprotectants inside the cell (Volk and Walters, 2006). However, the tissues overexposed to vitrification solutions may be severely impaired by the toxic nature of cryoprotectant agents and excessive dehydration.

Using vitrification method, the survival of cryostored *H. perforatum* shoot tips significantly increased with the enhancing dehydration and prolongation of the loading phase. However, the maximum mean recovery rate reaching nearly 70% was reported even if the freezable water up to 0.4% of the total water content (0.005 g freezable H_2_O g^-1^ DW) was present in the cryopreserved tissues (Bruňáková et al., 2011). In meristematic cells of *H. perforatum*, the incidence of small endothermal transitions proportional up to 3.5% of freezable water (0.05 g freezable H_2_O g^-1^ DW) was shown to have no lethal effects on the post-cryogenic recovery. This observation indicates an existence of other protective mechanisms preventing the freezing injury during cryopreservation.

## Post-Cryogenic Recovery in Relation to Freezing Tolerance in *Hypericum* Spp.

Along with the influence of external factors, the genetic predetermination of cold response affecting the post-cryogenic survival within the genus *Hypericum* should be highlighted. More than 450 *Hypericum* species have been found predominantly in a variety of biotopes connected to the temperate regions and high elevation in the tropics (Crockett and Robson, 2011). Apart from dehydration, osmotic and high-temperature stresses, low temperature represents a limiting factor for the species distribution as well. In general, plants adapt to freezing by the ‘tolerance’ or ‘avoidance’ strategies (Levitt, 1980). The freezing response is a complex mechanism reflecting the biology of water and its interactions with cellular components at low temperatures (Olien and Livingston, 2006). The cold-tolerant species can overcome sudden decreases of the temperatures below 0°C and improve their ability to survive potentially damaging temperatures after cold acclimation (Levitt, 1980). During the adaptation process, plants accumulate endogenous cryoprotective substances that prevent cell injury by stabilizing the membranes and proteins under dehydration conditions, inhibiting the growth of ice crystals, preventing re-crystallization, interacting with other molecules to scavenge the reactive oxygen species (ROS), etc., (Gusta and Wisniewski, 2013). On the other hand, the freezing avoidance means that plants do not tolerate ice formation in their tissues and avoid crystallization, e.g., by supercooling.

### Freezing Tolerance Based on Frost-Killing Temperature LT_50_

The results concerning FT in the *Hypericum* genus are based on assessment of the extent of freezing injury performed by measurement of the electrolyte leakage. The FT of several *Hypericum* species was estimated according to the temperature at which 50 percent of the shoot tips were lethally damaged (LT_50_; Petijová et al., 2014). Among the studied taxa, *H. perforatum* L. and *H. kalmianum* were proved to be the most freezing-tolerant species tolerating the temperatures up to -9°C when acclimated at 4°C for 7 days. The capacity to increase the tolerance to low temperatures depends on several factors, e.g., the temperature and regime of acclimation as well as the quantity and intensity of light (Levitt, 1980; Monroy et al., 1993). In the model *H. perforatum*, a greater enhancement of the FT was observed upon exposure to gradually decreasing temperature from 22°C up to 4°C at a rate of 1°C day^-1^ (Bruňáková et al., 2015). Based on the three-fold depress of the LT_50_ value, *H. perforatum* was shown to possess the protective mechanisms associated with two major components: the constitutive FT expressed by LT_50_ = -5.6°C, and cold-acclimation capacity to increase FT reaching LT_50_ = -16.2°C. Considering the nearly cosmopolitan distribution of *H. perforatum* and the endemic occurrence of *H. kalmianum* in the cold area of USA and Canada adjacent to the Great Lakes and the Ottawa River (Robson, 1996), the extent of FT reflected the ecological demands of these species.

While the processes associated with FT are based rather on biochemical adaptations, the freezing avoidance is more connected to physical attributes of plants which determine the preferential sites of ice accumulation through the presence or absence of ice nucleators, anatomical ice barriers, lowering the freezing point or supercooling (Gusta and Wisniewski, 2013). However, freezing avoidance is only safe under conditions of mild frost that lasts short period of time, e.g., in tropical high mountains or during the spring frost periods (Wisniewski et al., 2009). As an example for the avoidance strategy in preventing freezing injury, *H. canariense* as the endemic species of Canary Island and Madeira (Robson, 1996) could be mentioned. No significant difference in the LT_50_ value was registered for the meristems excised from the control plants cultured under room temperature and plants exposed to 4°C showing the LT_50_ = -2.3°C and LT_50_ = -3.5°C, respectively (Bruňáková et al., 2015). However, *H. canariense* avoided freezing by supercooling when LT_50_ dropped up to -8.2°C.

The natural ability to acclimate represents a suitable alternative for preconditioning of plant tissues without any side effects that may result from the incubation on media supplemented with high concentration of cryoprotectans, such as sucrose, glycerol or ME_2_SO. Among herbaceous plants, cold exposure was shown to induce FT and improve the post-cryogenic recovery of cryopreserved meristems in *Solanum commersonii* (Folgado et al., 2015), *Lolium L.* and *Zoysia* Willd, grass cultivars (Chang et al., 2000), *Cynodon* spp. (Reed et al., 2006) and others. In *H. perforatum*, the exposure of plants to 4°C alone or in combination with ABA significantly enhanced the recovery of shoot tips cryopreserved by LS and PVS3 and immersed directly into LN (Bruňáková et al., 2014). It has been reported that FT represented by the LT_50_ were concurred with the geographical distribution and post-thaw survival of *Hypericum* species involved in the study of Petijová et al. (2014). While the highest post-cryogenic regeneration of 26% was registered for *H. kalmianum* as the most freeze-tolerant species, the cold-sensitive species growing in the tropical and subtropical areas such as *H. canariense* and *H. kouytchense* did not survive the cryogenic treatment at all.

### Structural and Physiological Markers of Freezing Tolerance

To obtain additional information on the FT in plants, the extent of freezing injury in terms of the integrity of photosynthetic apparatus has been intensively investigated at both, functional and structural levels (Rumich-Bayer and Krause, 1986; He et al., 2002; Su et al., 2015). Based on histological and TEM examination of the effect of freezing temperature on leaf tissue organization and chloroplast ultrastructure of seven *Hypericum* species differing in the LT_50_ values (Petijová et al., 2014; Stoyanova-Koleva et al., 2015), no plausible connection between the predicted FT and response to cryoinjury was observed. Under the experimental conditions including a 10-day ABA preculture, cryoprotection with PVS3 and rapid cooling by direct immersion into LN, the well-developed leaves with regularly structured mesophyll in post-cryogenic regenerants of *H. annulatum, H. tomentosum, H. rumeliacum, H. humifusum*, and *H. kalmianum* possessing a various extent of the FT were seen. A partial damage of chloroplasts ultrastructure was only observed in the freezing-tolerant *H. perforatum.* However, a remarkably increased thickness of the leaf assimilation parenchyma indicates a possible compensatory mechanism to overcome the low-temperature stress in that species (Stoyanova-Koleva et al., 2015).

Apart from the species-specific structural modifications observed in post-cryogenic regenerants of *Hypericum* spp., differences in the extent of oxidative damage are also documented at physiological level. Along with the natural enzymatic defense represented by ROS-scavenging enzymes that are known to accumulate in cryopreserved cells and tissues (Reed, 2014; Chen et al., 2015), the non-enzymatic physiological markers including chlorophylls, carotenoids, proline, phenolics, and flavonoids have been investigated (Skyba et al., 2010; Danova et al., 2012; Georgieva et al., 2014).

Molecular and biochemical aspects of the FT in *H. perforatum* were documented by Skyba et al. (2010, 2012). In post-cryogenic regenerants exhibiting deleterious damage of thylakoid membranes in a substantial proportion of chloroplasts, the elevated level of mRNA transcripts of catalase (*hp-cat*) and superoxide dismutase (*hp-sod*) genes followed by the increased activity of CAT and SOD enzymes were detected. In *H. rumeliacum*, markedly increased both, enzymatic and non-enzymatic antioxidant activities indicating an extensive ROS-production persisted during the regeneration phase for several months (Danova et al., 2012). While post-cryogenic regenerants of *H. rumeliacum* displayed several morphological and physiological deviations comprising shorter stems, decreased number of leaves, poorly developed root system and slower growth rate (Georgieva et al., 2014), no substantial modifications in the developmental pattern of *H. perforatum* were observed (Skyba et al., 2012). When compared with *H. rumeliacum*, the ability of *H. perforatum* to eliminate the phenotypic expression of an increased oxidative status, substantially lower LT_50_ value and increased post-cryogenic regeneration potential (Petijová et al., 2014) indicates *H. perforatum* as more tolerant to the freezing process.

On the other hand, no significant differences in the physiological state of post-cryogenic regenerants in another *Hypericum* representative – *H. tetrapterum –* were noticed (Georgieva et al., 2014). Although the lower degree of oxidative damage expressed by both, enzymatic and non-enzymatic physiological markers might suggest that the species is more tolerant to freezing, the potential of the species to withstand cryogenic treatment was much reduced when compared to *H. perforatum.* Although a successful post-cryogenic regeneration of *H. tetrapterum* was reported by Georgieva et al. (2014), the recovery of this species after cryopreservation was not confirmed in our laboratory under the same experimental conditions (Bruňáková et al., 2014; Petijová et al., 2014).

Apart from the inter-specific variation in avoiding the freezing injury among the *Hypericum* species (Petijová et al., 2012), a considerable intra-specific variability in the tolerance to cryogenic treatment using the physiological markers malondialdehyde (MDA), CAT, SOD, H_2_O_2_, total free proline, carotenoid, and hypericins content was reported (Skyba et al., 2010). In addition to the differences in biochemical status of the individual genotypes of *H. perforatum* before and after cryostorage, several molecular techniques including RAPD fingerprinting and VNTR minisatellite analysis were used to assess the genetic background for genotype-determined variability (Urbanová et al., 2006; Skyba et al., 2010; Skyba et al., 2012). While a marked variation within the species was repeatedly confirmed, no differences in RAPD/VNTR amplification profiles of the analyzed control and cryopreserved pairs of plantlets were reported (Skyba et al., 2012). However, the data concerning DNA primary structure analyses based on RAPD and minisatellite markers should be interpreted with regard to only a small fraction of diploid *H. perforatum* genome they reveal which is estimated to represent 0.003 and 0.001% of the genome, respectively. The genetic integrity was proved at cytogenetic level by chromosome counts and ploidy level in recovered plants of *H. perforatum* which were shown to preserve the original chromosomal number (2n = 2x = 16; Urbanová et al., 2006).

In general, an increased oxidative status referring to a cryoinjury primarily denotes a reduced tolerance of the species toward the freezing. However, activation of antioxidant mechanism might reflect genetically predetermined capability of the species to detoxify the ROS. Therefore, the overall tolerance of the plant species to low-temperature stress accompanying cryopreservation seems to be a multifactorial trait and should be evaluated in a complex manner.

## Is Cold Stress a Possible Elicitor of Naphthodianthrones Biosynthesis?

In higher plants, the products of secondary metabolic pathways mediate plant adaptation to changing environment and function as the signal molecules during ontogenesis (Broun, 2005; Zhao et al., 2005). The cold response of the overwintering plants consists in three consecutive phases involving: (i) exposure to temperatures above freezing ranging from 2 to 5°C, (ii) exposition to mild freezing from -2 to -5°C, and (iii) post-freezing recovery (Li et al., 2008). As a part of cold acclimation at above-zero temperatures, plant metabolism diverts to synthesize various metabolites involving saccharides, saccharide alcohols, low-molecular weight nitrogenous compounds proline and glycine-betaine, cold-regulated (COR) proteins, antioxidant enzymes, endogenous jasmonates, phenolics, flavonoids, polyamines and other substances exhibiting cryoprotective characteristics (Ramakrishna and Ravishankar, 2011; Espevig et al., 2012; Bhandari and Nayyar, 2014). During the second phase, the full degree of tolerance is achieved by the exposition of plants to mild freezing which is commonly associated with ice formation in apoplast and dehydration of plant cells (Steponkus and Lynch, 1989). In the post-freezing phase, the plant undergoes thawing, rehydration of the cells, and restoration of cell structures and functions (Li et al., 2008).

Naphthodianthrones are secondary metabolites known to be used by plants for defense (Kainulainen et al., 1996). It has been found that variability in the content of naphthodianthrones including hypericin, pseudohypericin and their protoformes (so-called ‘total hypericins’ or ‘total naphthodianthrones’) is determined genetically, and can be modulated by environmental parameters such as light conditions and temperature (Kirakosyan et al., 2004). In the context of temperature stress, the yield of total naphthodianthrones in *H. perforatum* was found to be positively influenced by enhancing the temperature up to 35°C (Zobayed et al., 2005). In response to low temperatures, the accumulation of hypericins was found to depend predominantly on the physiological status of the plants as well as on the cold treatment applying: (i) cold acclimation, (ii) cold-shock caused by subfreezing at -4°C and (iii) cryopreservation procedure.

With an exception of *H. tetrapterum*, the total hypericin content was unchanged or decreased upon exposure to 4°C for 7 days in several other *Hypericum* species, including *H. humifusum*, *H. annulatum, H. tomentosum*, and *H. rumeliacum* (Petijová et al., 2014). Exposure of plants to gradual temperature decrease significantly lowered the amount of total hypericins in the vegetative parts of the treated plants (Petijová et al., 2014; Bruňáková et al., 2015). Considering the fact that the period of cold acclimation of *H. perforatum* was shown to be accompanied by a decrease in the total water content (Bruňáková et al., 2011), the drop in the content of hypericins might be influenced by the increase in DW in the cold-acclimated plants. Similarly, during the period of cold acclimation of *Arabidopsis* ecotype Columbia plants, the rise of DW accompanied with the accumulation of sugars and proline was observed (Wanner and Junttila, 1999).

On the other side, a beneficial effect of the cold shock on the stimulation of naphthodianthrones biosynthesis in *H. perforatum* was shown by Bruňáková et al. (2015). In contrast to the unchanged level of total hypericins in cold-acclimated *H. perforatum* plants subjected to -4°C, the 48-h subfreezing treatment of the controlled, non-acclimated plants resulted in the 1.6-fold increase of the total naphthodianthrones content. In the same study, the significant enhancement of the carotenoids in non-acclimated *H. perforatum* plants serves as another evidence for the stimulatory effect of the cold-shock treatment.

**FIGURE 1:**
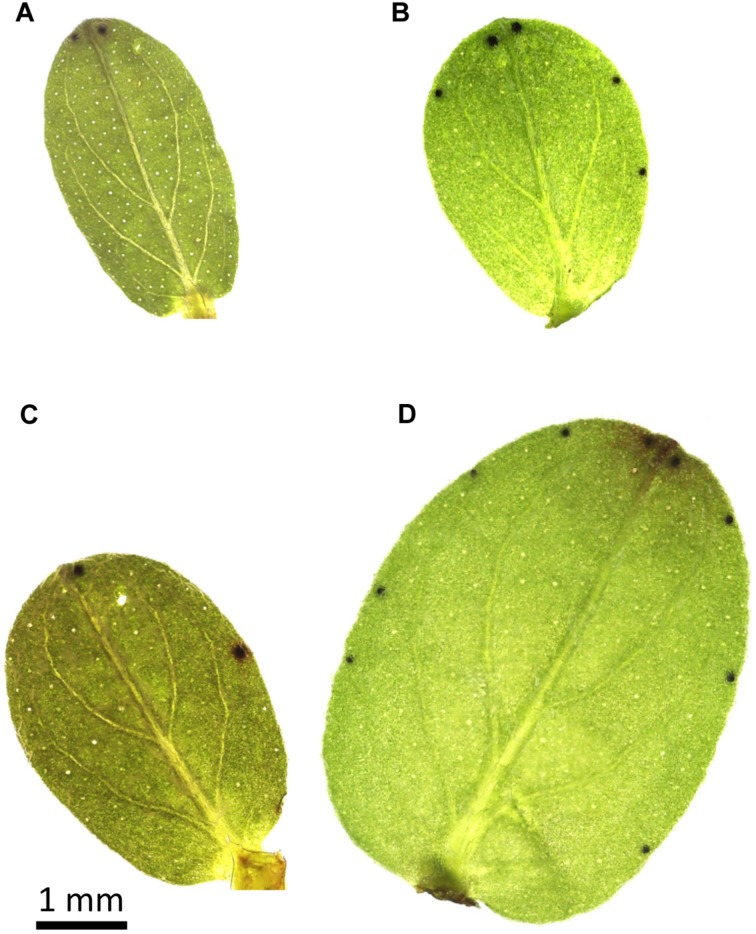
**The number and distribution of dark nodules on the first pair **(A,B)** and fourth pair **(C,D)** of leaves of *in vitro* grown *Hypericum tomentosum* control plants **(A,C)** and shoots regenerated after cryopreservation **(B,D)****.

It has been evidenced that total hypericins amount in the post-cryogenic regenerants depended on the physiological status of the explants entering the cryopreservation that could be manipulated by the pre-cryogenic conditions, e.g., the preconditioning with ABA or cold treatment (Danova et al., 2012). Based on the HPLC analyses, the enhanced total hypericins content was seen in *H. rumeliacum* shoots regenerated from the cryopreserved shoot tips that were exposed to ABA pre-treatment for the period of 7 days. In contrast, a shorter exposure did not show any stimulatory effect on hypericins biosynthesis (Danova et al., 2012; Bruňáková et al., 2014; Georgieva et al., 2014; Petijová et al., 2014). On the other side, the higher level of total hypericins reaching 3.1 and 1.6-fold increase, respectively, was found in *H. perforatum* and *H. rumeliacum* regenerants initiated from the cryopreserved shoot apices isolated from cold-acclimated plants (Bruňáková et al., 2014). Although the induction effect of cryo-environment on the formation of dark nodules is ambiguous (Petijová et al., 2014), an increased number of these hypericins reservoirs on the leaves in post-cryogenic regenerants of some *Hypericum* species was evidenced (**Figure 1**). Considering the fact that accumulation of hypericins and total phenolics in *Hypericum* species was reported to be positively influenced by extreme conditions of the higher altitudes including low temperatures (Rahnavard et al., 2012), the enhancement of the constitutive FT by a process of cold acclimation prior to cryopreservation treatment may potent biosynthesis of these compounds as a part of natural plant defense. Along with naphthodianthrones, the cold stress was shown to enhance the flavonoids content in the post-cryogenic *H. rumeliacum* and *H. tetrapterum* regenerants (Danova et al., 2012).

## Conclusion

Apart from the model *H. perforatum*, several *Hypericum* species involving *H. rumeliacum*, *H. kalmianum*, *H. annulatum*, *H. humifusum*, *H. tomentosum*, *H. tetrapterum*, *H. pulchrum*, *H. richeri* ssp. *Transsilvanicum*, and *H. umbellatum* have been successfully cryopreserved applying encapsulation-dehydration, droplet-vitrification, slow cooling and vitrification methods. Over the last 20 years, an intensive research has been conducted on the evaluation of critical factors affecting post-cryogenic survival. Among them, the physiological status of donor plants as a source of shoot tips as well as external parameters, e.g., optimal dehydration of meristems in course of slow cooling and minimization of the intensity of phase transitions during vitrification were identified.

The protection effects of ABA were proved at physiological and structural levels by lowering the water content including the proportion of freezable water, preservation of the meristematic tissue integrity and stabilization of internal membrane system of photosynthetic apparatus. Although some differences in intra- and interspecific responses in relation to the exposure time and cooling regime were observed, application of ABA, sucrose and/or mannitol during the pre-culture treatment positively influenced the recovery after cryopreservation.

On the other side, applying the uniform cryopreservation conditions to *Hypericum* species of various provenances resulting in a broad variability in post-cryogenic recovery pointed out the significance of genetically predetermined ability to resist the cryogenic injury. In the freezing-tolerant species, the exposure of donor plants to temperature of 4°C prior to cryoprotection was shown to completely substitute the effects of ABA and resulted in an improved survival and regeneration of shoots from the cryopreserved meristems. Applying a slow-cooling regime, the improved survival could be attributed to higher integrity of meristematic domes at lower rates of cooling. Using the vitrification-based protocols, the recovery rates depended on length of the loading phase which was proved to be critical for survival balancing the dehydration and toxic effects of cryoprotectants.

Apart from structural changes in the leaf tissue organization and chloroplast ultrastructure, several physiological markers indicating the freezing injury in post-cryogenic regenerants were assessed. The increased activity of ROS-scavenging enzymes CAT, SOD indicated that higher oxidative stress persisted in regenerated shoot tissues of some *Hypericum* species in spite of the fact that no substantial alteration of the phenotype was present. The increased content of proline, green pigments, carotenoids, phenolics, and flavonoids observed in the shoot tissues regenerated from cryopreserved meristems of *Hypericum* spp. are in consent with an overall plant response to extreme abiotic conditions. Similarly, an enhanced accumulation of naphthodianthrones hypericin, pseudohypericin, and their protoforms under low-temperature stress could be considered as a part of natural mechanisms of a systemic defense in the freezing-tolerant hypericin-producing species.

In conclusion, to achieve an improved post-cryogenic survival along with maintenance of the physiological functions and biochemical potential of the post-cryogenic regenerants of *Hypericum* spp., the modifications of the cryconservation protocol with respect to the species-specific plant responses to low-temperature treatment in the context of predetermined FT are inevitable.

## Author Contributions

This review was conceived and supervised by EČ. The concept of the review was worked out by both EČ and KB. Author of the manuscript draft, photographs and figure image was KB. The manuscript was revised and finally approved by EČ.

## Conflict of Interest Statement

The authors declare that the research was conducted in the absence of any commercial or financial relationships that could be construed as a potential conflict of interest.
